# Genome-Wide Association Study of Vascular Bundle-Related Traits in Maize Stalk

**DOI:** 10.3389/fpls.2021.699486

**Published:** 2021-07-16

**Authors:** Yunxiao Zheng, Peng Hou, Liying Zhu, Weibin Song, Han Liu, Yaqun Huang, Hong Wang, Jinjie Guo

**Affiliations:** ^1^State Key Laboratory of North China Crop Improvement and Regulation, Hebei Sub-center for National Maize Improvement Center, College of Agronomy, Hebei Agricultural University, Baoding, China; ^2^Key Laboratory of Crop Physiology and Ecology, Ministry of Agriculture and Rural Affairs, Institute of Crop Sciences, Chinese Academy of Agricultural Sciences, Beijing, China; ^3^State Key Laboratory of Agrobiotechnology, National Maize Improvement Center, Department of Plant Genetics and Breeding, China Agricultural University, Beijing, China; ^4^Institute of Botany, Chinese Academy of Sciences, Beijing, China

**Keywords:** maize, stalk, vascular bundle, genome-wide association study, candidate genes

## Abstract

The vascular bundle plays an important role in nutrient transportation in plants and exerts great influence on crop yield. Maize is widely used for food, feed, and fuel, producing the largest yield in the world. However, genes and molecular mechanism controlling vascular bundle-related traits in maize have largely remained undiscovered. In this study, a natural population containing 248 diverse maize inbred lines genotyped with high-throughput SNP markers was used for genome-wide association study. The results showed that broad variations existed for the vascular bundle-related traits which are subject to genetic structure and it was suitable for association analysis. In this study, we identified 15, 13, 2, 1, and 5 SNPs significantly associated with number of small vascular bundle, number of large vascular bundle, average area of single small vascular bundle, average area of single large vascular bundle, and cross-sectional area, respectively. The 210 candidate genes in the confidence interval can be classified into ten biological processes, three cellular components, and eight molecular functions. As for the Kyoto Encyclopedia of Genes and Genomes analysis of the candidate genes, a total of six pathways were identified. Finally, we found five genes related to vascular development, three genes related to cell wall, and two genes related to the mechanical strength of the stalk. Our results provide the further understanding of the genetic foundation of vascular bundle-related traits in maize stalk.

## Introduction

Maize is the most widely planted crop in the world and provides a large part of food for animals and human as well as materials for deep processing and energy. According to the forecast of the United Nations, the world’s population will exceed nine billion and global demand for maize will double (Ray et al., 2013) by 2050. In order to provide living space for the growing population, the cultivated land area has shrunk year by year worldwide. Thus, it is of great significance to increase the yield per unit area of maize.

Vascular bundles consist of xylem and phloem, two differentiated conductive tissues, and undifferentiated cambial or procambial stem cells. Vascular bundles provide mechanical support for plants. In maize, appropriate strength and tenacity would help the plant stand against winds and decrease the loss caused by lodging. Moreover, vascular bundles function as “flow” in the “source-flow-sink” system and transport photoassimilates produced by leaves (“source”) to the fruit (“sink”) and transfer water and salt from roots to the whole plants. Previous studies have observed significant correlation between the vascular bundle system and maize yield (Housley and Peterson, 1982; Nátrová, 1991).

Many studies have been carried out on the genes related to vascular bundle system in plants. In Arabidopsis, genes involved in vascular bundle patterning have been identified, such as *MP*, *PHB*, *PHV*, *AtHB15*, and *REV* (Hardtke and Berleth, 1998; McConnell et al., 2001; Zhong and Ye, 2004; Du and Wang, 2015). In crops, quantitative trait loci (QTL) for vascular bundle-related traits have been identified in tomato (Coaker et al., 2002), wheat (Sang et al., 2010), and rice (Bai et al., 2012; Zhai et al., 2018; Fei et al., 2019). Notably, genes that affect the vascular bundle system in rice have been reported, such as *APO1*, *ABV*, *DEP1*, and *NAL1* (Qi et al., 2008; Terao et al., 2010; Fujita et al., 2013; Fei et al., 2019). However, the functional genes have not been fully discovered and the molecular mechanism of how the vascular bundle system influences the crop yield has remained largely unknown.

Progresses have been made in the study of maize vascular bundles system. Sakaguchi and Fukuda (2008) used the basal leaves to observe the continuous process of vascular bundle cell development and accordingly divided the longitudinal vein development of maize leaves into five stages. Feng et al. (2014) found that planting density significantly affected the structure of maize stem vascular bundles and that, with the increase in planting density, the number of large and small vascular bundles in the stem decreased. In addition, the number of large vascular bundles and that of total vascular bundles differed significantly between different density treatments. Xu et al. (2017) found that plant growth regulator EDAH significantly increased the number and area of vascular bundles. However, earlier studies mainly focused on the development and micro-structure of vascular bundles, and little is known on the functional genes regulating the vascular bundle system in maize, and the molecular mechanism on the vascular bundle-related traits in maize has remained largely blank. Huang et al. (2016) used a large maize-teosinte experimental population to perform a high-resolution QTL mapping for the number of vascular bundle in the uppermost internode of maize stem and validate the effect of one QTL *qVb9-2* on chromosome 9.

In recent years, genome-wide association study (GWAS) has become an efficient tool to capture functional genes and favorable haplotypes for interested traits in maize (Tian et al., 2011; Li et al., 2013, 2019; Wang et al., 2016). For maize, due to the release of the B73 reference genome, GWAS application in agricultural traits of maize provides useful reference for revealing the phenotypic traits diversity and genetic architecture of vascular bundles in maize stalk. In this study, we performed GWAS for vascular bundle system using a natural population consisting of 248 maize inbred lines with abundant genetic diversity in 2017, 2018, and 2019. And the candidate genes adjacent to the significant SNPs were identified. This study lays the foundation for understanding the genetic architecture of vascular bundle-related traits in maize.

## Materials and Methods

### Plant Materials

A natural population panel containing 248 diverse maize inbred lines was used as research materials provided by the China Agricultural University and National Maize Improvement Center of China. The 248 maize inbred lines panel included not only some excellent back bone elite inbred lines in China but also some high-quality inbred lines introduced from abroad. The detailed information on this natural population can be found in Supplementary Table S1 (Yang, 2016). For all the 248 maize inbred lines, a randomized block design with two replications was used in this study. Each material inbred was planted in a plot of two 3.0-m-long rows with 0.60-m-inter-row space, using a population density of 75,000 plants per hectare at the Experimental Station of Hebei Agricultural University in Baoding (115.48° and 38.85°) in the summer of 2017, 2018, and 2019 and the Experimental Station of Shijiazhuang (115.12° and 37.54°) in the summer of 2017 and 2018, respectively. All the maize plants were in-followed standard local field management using local maize tillage methods throughout the whole growth periods.

### Phenotypic Evaluation

One week after pollination, three individual plants with typical growth were selected for each inbred as three biological replicates. For each replicate, cross-section slices from the uppermost internode were manually made. The slices were stained with 5% (g/ml) m-trihydroxybenzene and concentrated hydrochloric acid (Huang et al., 2016). Then, the stained slices were imaged by the Zeiss Axioskop 40 microscope (Germany). Zen (blue edition) 2012 image processing software was used to collect data of vascular bundle-related traits, including the number of small vascular bundle (NSVB), the number of large vascular bundle (NLVB), average area of single small vascular bundle (ASVB), average area of single large vascular bundle (ALVB), and cross-sectional area (CSA). The vascular bundles of stalk are divided into two categories: small vascular bundles and large vascular bundles. The small vascular bundles are generally located in the 1~2 layer of the edge of the tissue, with relatively close arrangement, the area is small and some small vascular bundles are not fully developed; the large vascular bundles are located in the organization, loose arrangement, relatively large area, and complete structural development. The vascular bundle area and CSA were calculated in terms of near-ellipse S=πab/4 (a and b represent the major and minor axes of the ellipse, respectively; Yang, 2016).

### Phenotypic Analysis

Phenotypic data were processed with the Microsoft Excel 2010, and descriptive statistical analysis and ANOVA were carried out with the IBM SPSS statistics v21.0 software (George and Mallery, 2013). The broad-sense heritability ($h^{2}$) for each trait was estimated using the formula: $h^{2}=\sigma_{g}^{2}/(\sigma_{g}^{2}+\sigma_{gy}^{2}/r+\sigma_{e}^{2}/yr$), as described by Knapp et al. (1985), where $\sigma_{g}^{2}$ stands for the genetic variance, $\sigma_{gy}^{2}$ stands for genotype-by-environment interaction variance, $\sigma_{e}^{2}$ for error variance, y for the number of environments, and r for the number of replications. Correlations analysis was performed using the “Performance Analytics” package in R. The boxplot was drawn using “ggplot2” package in R. The best linear unbiased prediction (BLUP; Henderson, 1975) values using the mixed linear model of the “lme4” package in R was calculated for each trait across five environments and adopted as the phenotypic values in the subsequent genome-wide association study.

### Genotyping

The DNA of 248 maize inbred lines was extracted from fresh leaves with the CTAB method (Murray and Thompson, 1980). The sequencing libraries of 248 inbred lines were constructed and sequenced by genotyping-by-sequencing, and the qualified library was sequenced by the second-generation sequencer Illumina Hiseq2000 (Elshire et al., 2011). The derived short reads were compared to the reference genome of the second version of B73 by BWA software, and the SNPs were called by the SAMtools software to obtain the preliminary SNP markers. A total of 10,63,728 initial SNPs were obtained through strict sequencing data comparison and SNP-calling. Then, the SNPs with missing rate over 80% and minor allele frequency less than 5% were removed. Finally, a total of 83,057 SNPs were achieved and used in GWAS (Lai, 2017). The population structure of 248 inbred lines was detected by the Admixture 1.3 software (Chakraborty and Weiss, 1988), which was divided into five subpopulations: Lancaster, Lvda Red Cob, Tangsipingtou (TSPT), P group, Reid, and a mixed group. The Q model of population structure was obtained, and the K model of kinship was obtained by using the Analysis-Kindship in Tassel 5.0 software (Du et al., 2018).

### Genome-Wide Association Analysis

GWAS was performed to identify the SNPs significantly associated with vascular bundle-related traits using 83,057 SNPs and the BLUP values of the 248 maize inbred lines. GWAS was performed with the FarmCPU model implemented in the GAPIT package in the R software, taken both K and Q matrix into account to decrease spurious association (Liu et al., 2016). Then, we used the GEC software (Li et al., 2012) to calculate effective marker number ($N_{e}$), and the significance threshold was set as *p* ≤ 1/$N_{e}$.

### Identification of Candidate Genes

Linkage disequilibrium (LD) decay of the same population was analyzed in the previous studies (Li et al., 2020; Liu et al., 2020). The studies showed that the LD decay distance for this natural population panel was 120 kb (*r*^2^ = 0.1). Then, we searched the flanking 120 kb upstream and downstream of each significant loci for candidate genes according to B73 reference genome version v2. The annotation of candidate genes was obtained from the MaizeGDB and NCBI.^1^ The candidate genes were uploaded to GENE ONTOLOGY Web site^2^ for GO analysis. The KOBAS 3.0 Web site^3^ was used to perform the Kyoto Encyclopedia of Genes and Genomes (KEGG) pathway enrichment analysis.

## Results

### Diversity and Heritability of Vascular Bundle-Related Traits

**Figure 1:**
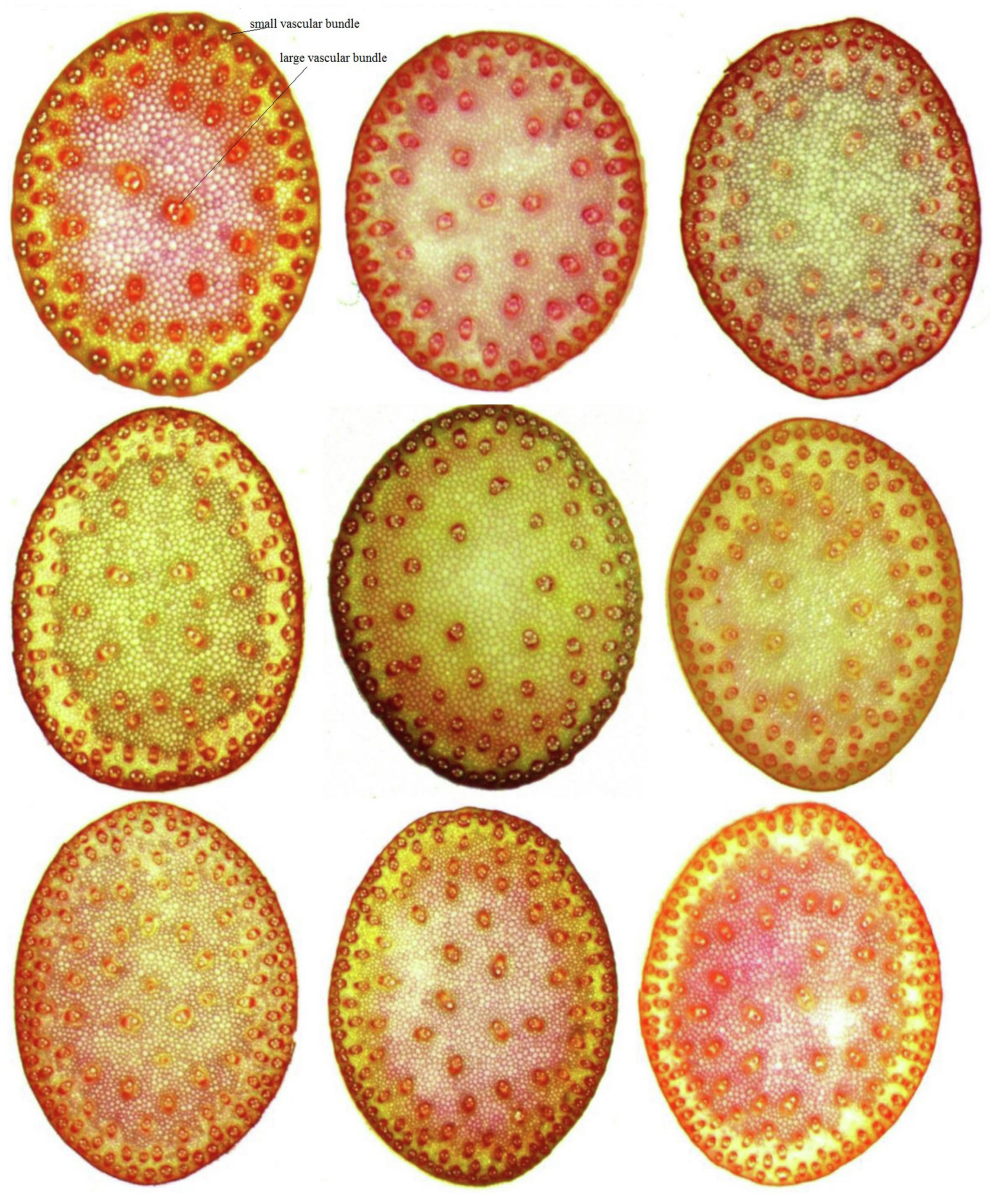
Vascular bundle structure of different maize inbred lines.

**Figure 2:**
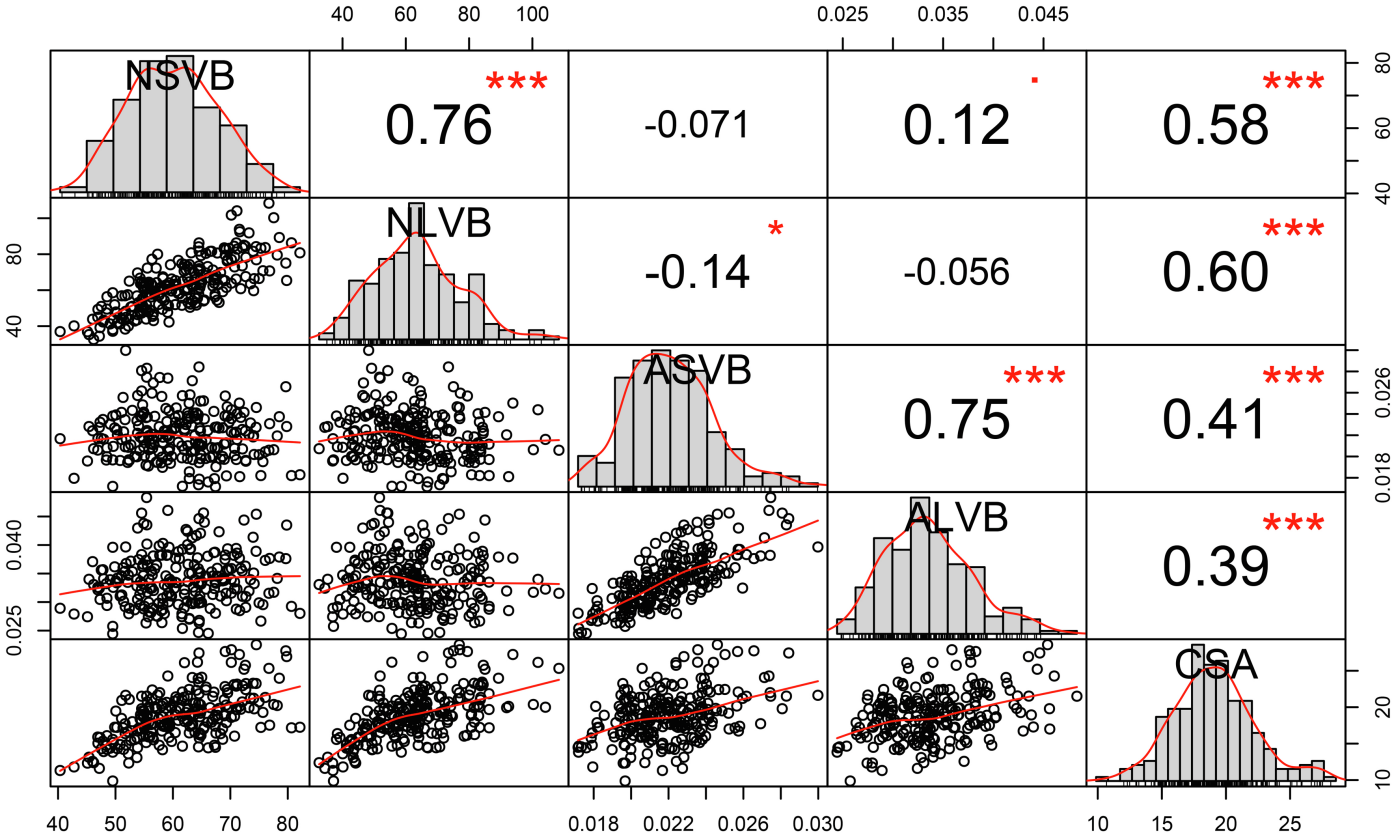
Correlation analysis of BLUP data for vascular bundle-related traits. NSVB, number of small vascular bundle; NLVB, number of large vascular bundle; ASVB, average area of single small vascular bundle; ALVB, average area of single large vascular bundle; and CSA, cross-sectional area. *Significant at 0.05 probability level. ***Significant at 0.001 probability level.

Substantial variation was observed for vascular bundle-related traits in different maize inbred lines (Figure 1). The statistical data for NSVB, NLVB, ASVB, ALVB, and CSA across the five environments showed that all five vascular bundle-related traits showed a normal distribution (Figure 2). The phenotype range of the above five traits was 17.933∼22.038, 26.106∼33.111, 26.283∼52.668, 27.308∼41.249, and 28.363∼37.095 (%), respectively (Table 1), exhibiting enriched genetic variation.

**Table 1:** Statistical analysis of vascular bundle-related traits in different environments.

Trait	Environment	Range	Mean	SD	Skewness	Kurtosis	CV (%)
NSVB	2017BD	31.000–87.000	57.509	10.946	0.198	−0.422	19.034%
	2017SJZ	34.000–96.000	62.141	11.261	0.308	−0.330	18.121%
	2018BD	32.000–87.000	57.182	10.254	0.187	−0.005	17.933%
	2018SJZ	36.000–98.000	61.825	10.602	0.306	−0.015	17.149%
	2019BD	31.000–110.000	66.525	14.660	0.484	−0.128	22.038%
NLVB	2017BD	27.000–114.000	61.416	16.226	0.378	−0.051	26.420%
	2017SJZ	28.000–136.000	66.130	19.043	0.567	0.325	28.796%
	2018BD	24.000–113.000	60.115	15.694	0.372	0.178	26.106%
	2018SJZ	28.000–116.000	61.043	16.558	0.495	0.138	27.126%
	2019BD	18.000–151.000	61.916	20.501	0.548	0.42	33.111%
ASVB	2017BD	0.008–0.043	0.021	0.006	0.653	0.641	28.891%
	2017SJZ	0.009–0.032	0.018	0.005	0.556	−0.248	26.922%
	2018BD	0.010–0.037	0.020	0.005	0.666	0.277	26.283%
	2018SJZ	0.009–0.040	0.021	0.006	0.763	0.571	27.582%
	2019BD	0.005–0.089	0.030	0.016	0.710	0.074	52.668%
ALVB	2017BD	0.011–0.070	0.033	0.010	0.783	0.941	31.478%
	2017SJZ	0.007–0.054	0.024	0.007	0.665	0.741	29.940%
	2018BD	0.014–0.069	0.031	0.010	0.944	0.858	30.894%
	2018SJZ	0.013–0.059	0.029	0.008	0.448	0.020	27.308%
	2019BD	0.104–0.111	0.053	0.022	0.297	−0.638	41.249%
CSA	2017BD	6.960–36.729	19.473	5.523	0.381	0.100	28.363%
	2017SJZ	6.875–33.609	16.173	4.685	0.877	1.197	28.968%
	2018BD	6.079–31.339	15.382	4.475	−0.733	0.547	29.091%
	2018SJZ	7.558–34.656	17.365	4.729	0.523	0.206	27.232%
	2019BD	5.093–60.520	25.520	9.584	0.544	0.076	37.095%

BD, Baoding; SJZ, Shijiazhuang. NSVB, NLVB, ASVB, ALVB, and CSA.

In the analysis of variance for the five traits, highly significant variations for genotypes (G), environments (E), and genotype-by-environment interaction were found (Table 2). This indicates the important roles of both genotypes, environment, and G × E interaction. The broad-sense heritability for NSVB, NLVB, ASVB, ALVB, and CSA across the five environments in the 248 inbred lines ranged from 46.49% (ASVB) to 91.52% (NLVB), indicating the predominant role of genetic factors for these traits (Table 2).

**Table 2:** ANOVA for vascular bundle-related traits.

Trait	*F*-value			
	Environment	Genotype	Environment^*^ Genotype	$h^{2}$(%)
NSVB	172.705^**^	30.161^**^	3.617^**^	88.01
NLVB	35.780^**^	41.275^**^	3.498^**^	91.52
ASVB	357.546^**^	7.879^**^	4.216^**^	46.49
ALVB	814.672^**^	8.331^**^	3.263^**^	60.83
CSA	440.955^**^	10.685^**^	2.488^**^	76.71

* Significant at 0.05 probability level.

** Significant at 0.01 probability level.

NSVB, NLVB, ASVB, ALVB, and CSA.

We calculated the BLUP values for each trait and observed significant correlations among them. CSA was positively correlated with NSVB (*r =* 0.58, *p* ≤ 0.001), NLVB (*r =* 0.60, *p* ≤ 0.001), ASVB (*r =* 0.41, *p* ≤ 0.001), and ALVB (*r =* 0.39, *p* ≤ 0.001). NSVB was positively correlated with NLVB (*r =* 0.76, *p* ≤ 0.001) and ASVB positively correlated with ALVB (*r =* 0.75, *p* ≤ 0.001). No significant correlations were detected between the vascular bundle area and the number of vascular bundles, except for the correlation between NLVB and ASVB (*r =* −0.14, *p* ≤ 0.05).

**Figure 3:**
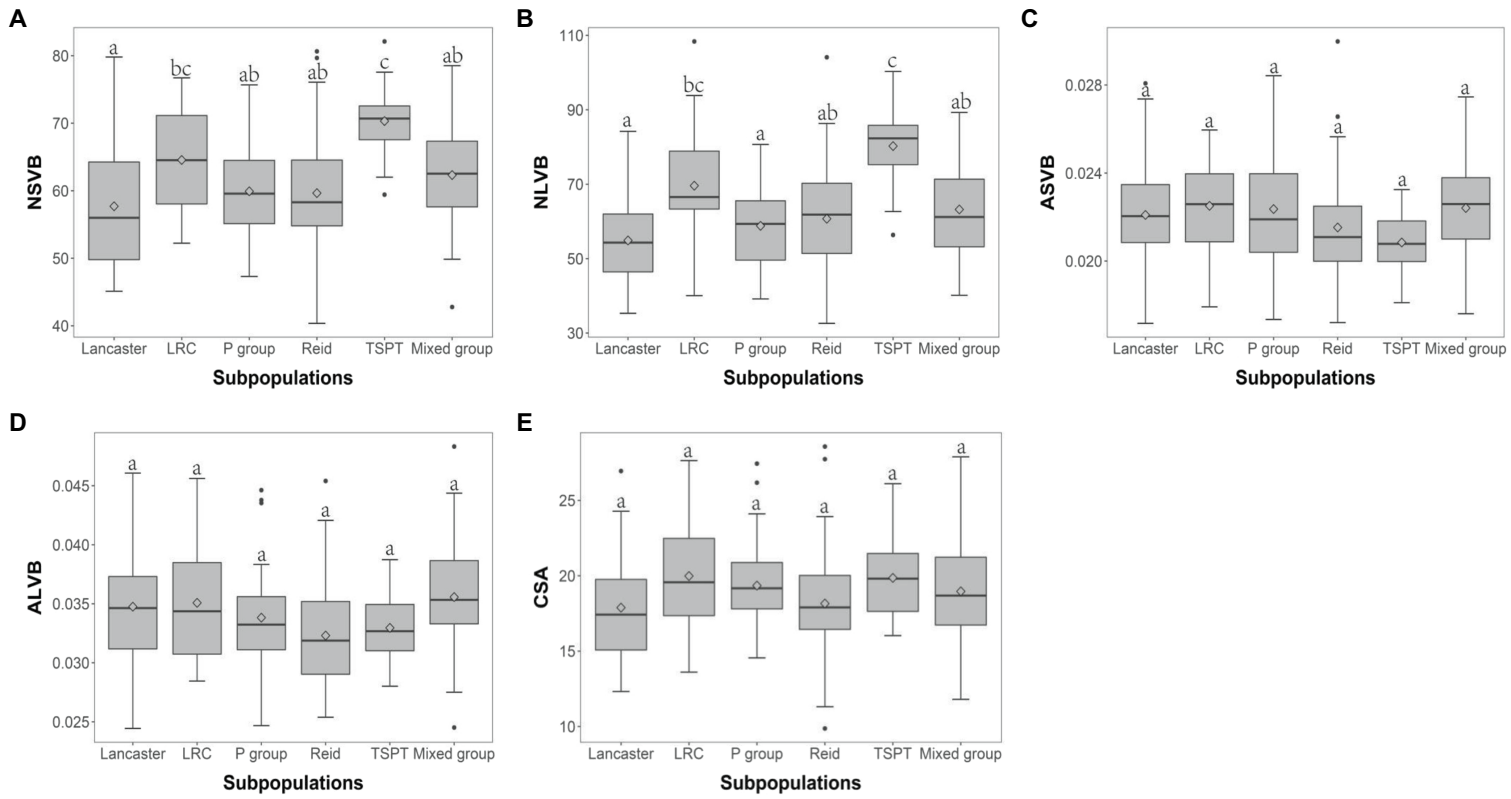
Boxplot of vascular bundle-related traits distribution in different subpopulations. ANOVA was applied to examine the difference of traits among subpopulations. Different numbers indicate significant difference at *p* ≤ 0.05. **(A)** NSVB; **(B)** NLVB; **(C)** ASVB; **(D)** ALVB; and **(E)** CSA.

The 248 inbred lines used in this study can be divided into five subpopulations and one mixed group, which are designated as Reid, Lancaster, TSPT, Lvda Red Cob (LRC), P group, and Mixed group, respectively (Liu et al., 2012, 2014). To investigate the effect of population structure on vascular bundle, the phenotypic variations of vascular bundle-related traits were compared between different subpopulations. For NSVB and NLVB, the means in TSPT subpopulation were significantly higher than the other subpopulations (Figures 3A,B). For ASVB, ALVB, and CSA, no significant difference was observed among the subpopulations, indicating that population structure has little effect on these traits (Figures 3C–E). In summary, the vascular bundle-related traits show broad variations which are subject to population structure.

### Genome-Wide Association Analysis

**Figure 4:**
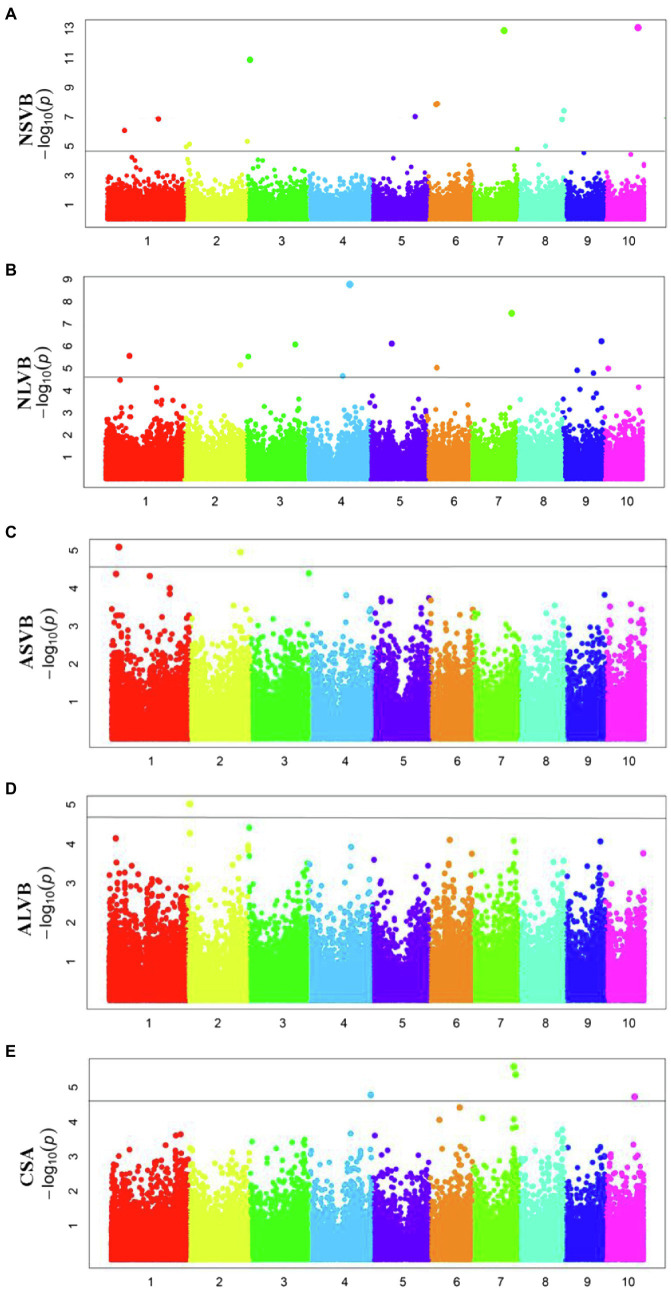
Manhattan plots of GWAS results for **(A)** NSVB; **(B)** NLVB; **(C)** ASVB; **(D)** ALVB; and **(E)** CSA.

To minimize the effect of environmental variation, BLUP values across five environments were used for GWAS. The effective number which was calculated using the GEC software was 40,705 and *p*-value which was recommended using the GEC software was 2.46E-5. Thus, the threshold is –log10 (2.46E-5) = 4.61. In total, we identified 15, 13, 2, 1, and 5 SNPs significantly associated with NSVB, NLVB, ASVB, ALVB, and CSA, respectively, based on –log10 *p* = 4.61 (Figure 4; Table 3). The single phenotypic variation explained value of NSVB, NLVB, ASVB, ALVB, and CSA varied in ranges of 0.68%~29.40, 1.09%~29.08, 6.01%~29.90, 16.27, and 21.05%~30.88%.

**Table 3:** Analysis of correlated SNP with vascular bundle-related traits.

Trait	Environment	SNP	Chromosome	Position	Allele	Bin	*P*-value	PVE
NSVB	BLUP	1_67048802	1	67048802	A/G	1.04	8.53E-07	4.41%
		1_196694835	1	196694835	A/C	1.06	1.38E-07	0.86%
		2_1716343	2	1716343	C/G	2.01	1.09E-05	10.72%
		2_14538772	2	14538772	A/C	2.02	7.17E-06	11.63%
		2_235478382	2	235478382	A/G	2.1	4.66E-06	3.01%
		3_8885082	3	8885082	C/T	3.03	1.29E-11	29.40%
		5_166864357	5	166864357	A/T	5.04	9.35E-08	6.12%
		6_29640289	6	29640289	A/C	6.01	1.41E-08	3.99%
		6_34222130	6	34222130	C/T	6.01	1.28E-08	0.68%
		7_121013955	7	121013955	A/G	7.02	1.34E-13	11.67%
		7_171236721	7	171236721	A/G	7.05	1.55E-05	1.85%
		8_103312474	8	103312474	C/T	8.03	9.72E-06	34.61%
		8_167013679	8	167013679	C/T	8.07	1.48E-07	10.84%
		8_173565720	8	173565720	C/T	8.09	3.81E-08	3.65%
		10_125107700	10	125107700	C/T	10.04	8.24E-14	18.80%
NLVB	BLUP	1_92518968	1	92518968	C/T	1.05	2.76E-06	5.42%
		2_215287936	2	215287936	A/G	2.08	7.17E-06	14.47%
		3_8885082	3	8885082	C/T	3.03	2.96E-06	21.35%
		3_188908168	3	188908168	G/T	3.06	8.46E-07	15.64%
		4_138195837	4	138195837	C/G	4.05	2.21E-05	19.44%
		4_165119210	4	165119210	G/T	4.06	1.70E-09	16.98%
		5_84576385	5	84576385	A/G	5.04	7.75E-07	0.01%
		6_39081558	6	39081558	A/G	6.01	9.38E-06	17.50%
		7_156667935	7	156667935	C/T	7.04	3.35E-08	29.08%
		9_55347902	9	55347902	C/T	9.03	1.23E-05	6.11%
		9_117308192	9	117308192	C/G	9.04	1.64E-05	1.09%
		9_148210107	9	148210107	C/T	9.06	6.07E-07	10.72%
		10_17676178	10	17676178	C/T	10.03	1.01E-05	19.42%
ASVB	BLUP	1_30348314	1	30348314	A/G	1.03	8.09E-06	29.90%
		2_197691099	2	197691099	A/G	2.07	1.11E-05	6.01%
ALVB	BLUP	2_10510180	2	10510180	C/T	2.02	9.46E-06	16.27%
CSA	BLUP	4_233224669	4	233224669	C/T	4.09	1.65E-05	30.88%
		7_156667935	7	156667935	C/T	7.04	2.48E-06	21.35%
		7_156667951	7	156667951	A/G	7.04	2.48E-06	21.35%
		7_164199008	7	164199008	C/G	7.04	4.26E-06	21.05%
		10_114005725	10	114005725	G/T	10.04	1.87E-05	22.89%

NSVB, NLVB, ASVB, ALVB, and CSA.

For five vascular bundle-related traits, we identified two pleiotropic loci that probably influenced more than one trait (hereinafter referred to as “co-loci”; Table 4). One located on Chromosome 3 (Chr3_8,885,082) was significantly associated with NSVB and NLVB, explaining from 21.35 to 29.40% of the phenotypic variation. The other one located at Chr7_156,667,935 was significantly associated with NLVB and CSA, explaining from 21.35 to 29.08% of the phenotypic variation.

**Table 4:** Co-loci of vascular bundle-related traits.

Number	Traits	SNP	Chr	Position	Allele	Bin	*P*-value	PVE (%)	Candidate Gene	RefGen_v2 Annotated Gene Description
1	NSVB	3_8885082	3	8885082	C/T	3.03	1.29E-11	29.40	GRMZM2G084125	Uncharacterized LOC100277349
	NLVB	3_8885082	3	8885082	C/T	3.03	2.96E-06	21.35	GRMZM2G083810	Heat shock protein18f
									GRMZM2G083763	Polynucleotide 5'-hydroxyl-kinase NOL9
									GRMZM2G083797	Probable inactive leucine-rich repeat receptor kinase XIAO
									GRMZM2G111846	Small subunit processome component 20 homolog
									GRMZM2G120271	Thioredoxin superfamily protein
									GRMZM2G144615	Uncharacterized LOC100384398
									GRMZM2G144668	1-aminocyclopropane-1-carboxylate oxidase
2	NLVB	7_156667935	7	156667935	C/T	7.04	3.35E-08	29.08	GRMZM2G051090	Uncharacterized LOC103633284
	CSA	7_156667935	7	156667935	C/T	7.04	2.48E-06	21.35	GRMZM2G050933	CYCD6
									GRMZM2G129973	Octicosapeptide/Phox/Bem1p family protein
									GRMZM2G153162	Eukaryotic translation initiation factor 4G
									GRMZM2G153454	Uncharacterized LOC100282357
									GRMZM2G153438	Uncharacterized LOC100383796
									GRMZM2G366977	Equilibrative nucleotide transporter 3

NSVB, NLVB, and CSA.

### Candidate Genes Associated With Significant SNPs

The physical locations of the significant SNPs were recorded according to the B73 RefGen_v2^4^ based on the LD decay. A total of 210 candidate genes with gene descriptions were found (Supplementary Table S2). The number of candidate genes involved in the vascular bundle-related traits of NSVB, NLVB, ASVB, ALVB, and CSA was 75, 55, 11, 23, and 19. The candidate genes were uploaded to GENE ONTOLOGY Web site (see footnote 2) for GO secondary classification.

**Figure 5:**
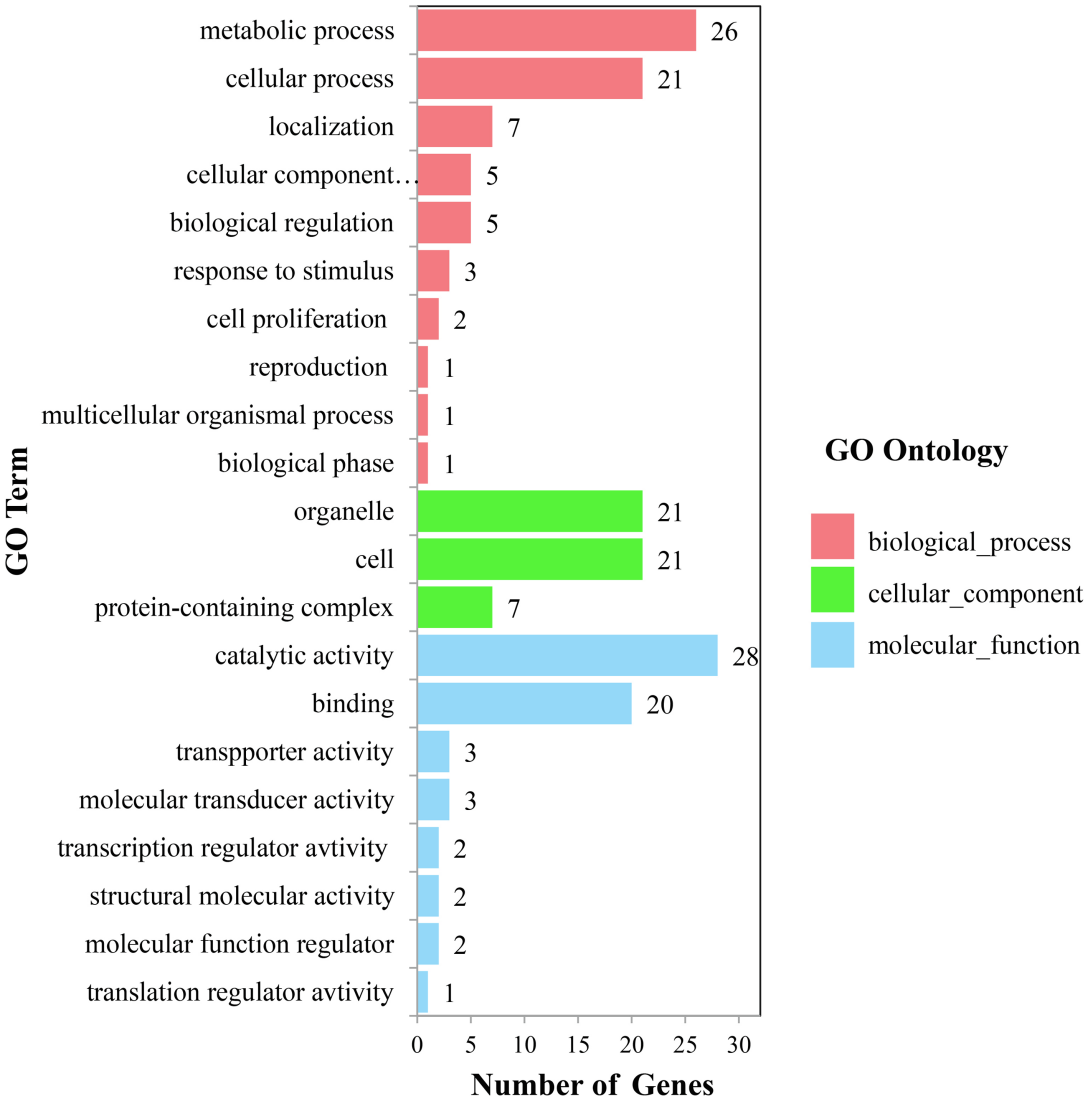
GO-second class of candidate gene.

**Figure 6:**
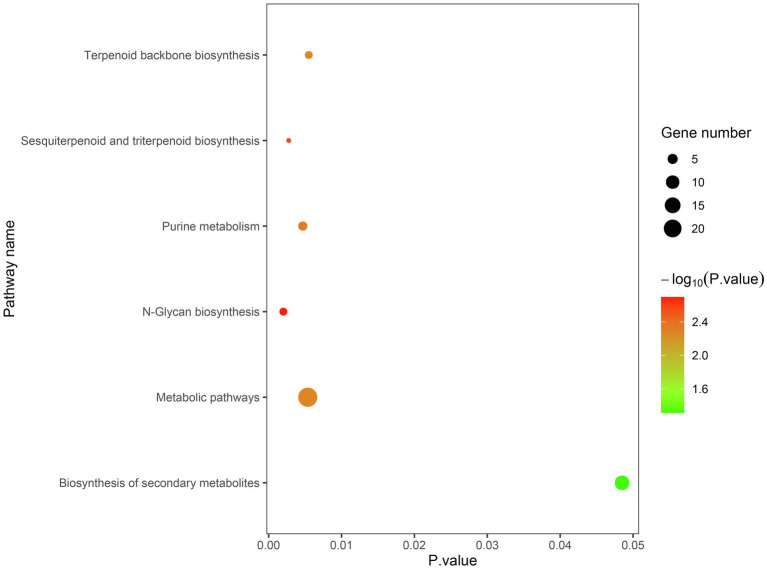
Analysis of KEGG pathway based on candidate genes.

The candidate genes can be classified into ten biological processes, three cellular components, and eight molecular functions. Among them, the candidate genes in biological processes are mainly concentrated in the metabolic process and the cellular process, the candidate genes in cellular component are mainly concentrated in cell and organelle, and those in molecular function are mainly concentrated in catalytic activity and binding (Figure 5). As for the KEGG analysis of the candidate genes, a total of six pathways were identified. These pathways included the metabolic pathways, biosynthesis of secondary metabolites, purine metabolism, N-Glycan biosynthesis, terpenoid backbone biosynthesis, and sesquiterpenoid and triterpenoid biosynthesis, which could be related to vascular bundle (Figure 6). Combined with functional annotation of the candidate genes, we finally found five genes related to vascular development, three genes related to cell wall, and two genes related to the mechanical strength of the stem (Table 5).

**Table 5:** Potential candidate genes for vascular bundle-related traits.

Trait	SNP	Bin	Candidate Gene	Gene ID	RefGen_v2 Annotated Gene Description
NSVB	2_14538772	2.02	GRMZM2G314396	542224	Calcium-dependent protein kinase
	8_103312474	8.03	GRMZM2G134687	103635646	NAC domain-containing protein 35
	8_173565720	8.09	GRMZM2G136400	103636631	CBL-interacting protein kinase 30
			GRMZM2G136410	103636630	CBL-interacting protein kinase 19
ALVB	2_10510180	2.02	GRMZM2G045596	100191933	Polygalacturonase QRT3
			GRMZM2G046070	100191885	Cinnamyl alcohol dehydrogenase 1
	7_164199008	7.04	GRMZM2G167520	100037799	Brittle stalk-2-like protein 6
			GRMZM2G167497	100037800	Brittle stalk-2-like protein 7
			GRMZM2G136971	103634382	Leucine-rich repeat extensin-like protein 3
	10_114005725	10.04	GRMZM5G831009	100216959	Purple acid phosphatase 22

NSVB and ALVB.

## Discussion

### Genetic Basis of Vascular Bundle-Related Traits

Studies have shown that plant vascular cells continuously connected shoot organs with roots from top to bottom, and the vascular bundles were polar, continuous, and internally aligned (Sawchuk and Scarpella, 2013). Due to the strong consistency between the upper vascular bundles and the lower ones, as well as the relatively clearer structure in upper vascular bundles after staining, vascular-related traits were collected from the uppermost internode in this study.

The five vascular bundle-related traits exhibited wide phenotypic variation with normal distribution. ANOVA showed that the genotype effects, environment effects, and interactive effects between the genotype and environment were both significant for five traits. The heritability ($h^{2}$) for these traits is very high, and ASVB was the least heritable of the traits included in this study. The correlations were observed among the area of vascular bundle and as were the number of vascular bundle. However, no significant differences were observed between the area of vascular bundle and the number of vascular bundles.

Subpopulations are often observed in natural maize populations. In this study, the maize panel was divided into five subpopulations, including TSPT, LRC, Lancaster, Reid, and P group (Lu et al., 2009; Yan et al., 2009; Yang et al., 2010, 2011). TSPT and LRC subpopulations were selected and improved from native resources in China (Ning et al., 2002; Feng et al., 2010; Xu, 2010), while Lancaster, Reid, and P groups were originated from the American germplasm resources (Guo et al., 2016). Therefore, the population structure may have imposed effects on the vascular bundles of maize. In this study, we observed no significant differences between subpopulations for CSA, ASVB, and ALVB, indicating that the area of vascular bundles is roughly analogous among each subpopulations. In contrast, the inbreds from TSPT and LRC possessed more vascular bundles, indicating that the vascular structure of native Chinese germplasm is more intense than that of American germplasm.

### Analysis and Comparison of Genome-Wide Association Analysis of Vascular Bundle-Related Traits

Vascular bundle-related traits are important traits of crops, which are not only highly correlated with crop yield traits, but also related to crop resistance. At the same time, some genes related to vascular bundle development have been cloned in model plants *Arabidopsis thaliana* and rice (Emery et al., 2003; Zhong and Ye, 2004; Terao et al., 2010; Fei et al., 2019). However, little is known about the functional genes in maize. On this ground, it is necessary to study the genetic basis of vascular bundle-related traits in maize.

In this study, a total of 36 different loci that are significantly associated with vascular bundle were detected. These loci may be key regions for the regulation of maize vascular bundle development. Among them, two “co-loci” were detected, each explaining over 20.00% of the phenotypic variation. This indicates that the loci had a major genetic effect and were less affected by the environment. Therefore, the two “co-loci” were the focus of the vascular bundle molecular design breeding or vascular bundle QTL map cloning work.

The 36 loci were compared with those of the previous studies. In this study, there were two loci which were consistent with the results of Yang (2016), one was located at Chr7_156,667,935 associated with NLVB and CSA; and the other was located at Chr7_156,667,951 associated with CSA. The two loci were very close, indicating that they may share the same functional gene. Du (2018) also found the locus which located at Chr7_156,667,935, indicating that it was stable and important for the development of maize vascular bundles. In addition, another two loci were consistent with the results of Du (2018), one was located at Chr10_125,107,700 associated with NSVB; and the other was located at Chr7_164,199,008 associated with CSA. Huang et al. (2016) performed a high-resolution QTL mapping for the number of vascular bundle in the uppermost internode of maize stem using a large maize-teosinte experimental population and validated the effect of one QTL *qVb9-2* on chromosome 9 and further fine mapped the QTL to a 1.8-Mb physical region. In this study, one locus controlling the number of vascular bundles was localized at Chr9_117.308192, about 1 Mb away from Huang et al. (2016) results.

### Candidate Genes Analysis

In this study, candidate genes were collected in the range of 120 kb upstream and downstream of the detected 36 significant association loci. A total of 210 candidate genes were listed, of which 64 genes could be used for GO analysis. These candidate genes involved a variety of biochemical metabolic pathways, such as metabolic process, cellular process, cell, organelle, catalytic activity, binding, and so on. Among them, GRMZM2G314396 encodes NAC domain-containing protein 35, NAC transcription factors are a specific class of transcription factors in plants, regulating the growth and development of plants, such as secondary wall and root growth, plant senescence, and so on. And they respond to a variety of abiotic and biotic stresses (Zhang et al., 2019). Kubo et al. (2005) used microarray analysis to find seven transcription factor proteins which containing the NAC domain associated with vascular development in Arabidopsis.

GRMZM2G314396, GRMZM2G136400, and GRMZM2G136410 are related to calcium. Calcium plays an essential role as the second messenger in cells in various signaling transduction pathways by developmental and environmental (Evans et al., 2001; Tuteja and Mahajan, 2007). GRMZM2G314396 encodes calcium-dependent protein kinase (CDPK) and the CDPKs are one of the well-known Ca2+−sensor protein kinases involved in environmental stress resistance (Harper and Harmon, 2005). AtCPK28, one of the CDPKs in Arabidopsis, has been reported to regulate plant stem elongation and vascular development by altering the expression of NAC transcription regulators and gibberellin homeostasis regulators (Matschi et al., 2013). GRMZM2G136400 and GRMZM2G136410 encode CBL-interacting protein kinase (CIPK). Calcineurin B-like proteins (CBLs) and their target proteins–the CIPKs have emerged in a key Ca^2+^-mediated signaling network in response to stresses in plants (Chen et al., 2011a). Lee et al. (2005) found that expression of AtCIPK14, one of the CIPKs in Arabidopsis, is restricted predominantly to the vascular tissues.

GRMZM2G046070 encodes cinnamyl alcohol dehydrogenase 1, which is the last enzyme in lignin monomer synthesis pathway. Previous studies have shown that lignin is an important component of almost all vascular plant intact cell walls (Battle et al., 2000; Zhong et al., 2000). Zhu et al. (2014) showed that *BnCAD1* expressed abundantly in the vascular bundle tissues in ramie. In addition, cinnamyl alcohol dehydrogenase was also found to relate to lodging in Arabidopsis (Jourdes et al., 2007), wheat (Ma, 2010; Chen et al., 2011b), rape (Huang et al., 2013), and so on.

Interestingly, we found some candidate genes related to the cell wall. As is known, cell elongation and cell wall thickening are involved in regulating lodging resistance in plants (Fan et al., 2018). Many studies have shown that vascular bundle-related traits affect the lodging resistance of crops, and vascular bundle-related traits are one of the breeding indexes for lodging resistance of crops (Bian et al., 2017; Hu et al., 2017). GRMZM2G045596 encodes polygalacturonase QRT3. Polygalacturonase, which can degrade pectin, is a plant cell wall structural protein (Abbott and Boraston, 2007). Xiao et al. (2017) overexpressed the *PGX2* gene in Arabidopsis, resulting in an increase in lignin in the stems of transgenic plants. GRMZM5G831009 encodes purple acid phosphatase 22. Purple acid phosphatases are members of the metallo-phosphoesterase family identified from a wide range of plants and play vital roles in modulating plant carbon and phosphorus metabolism, cell wall synthesis, and so on. NtPAP12 of tobacco participates in the biosynthesis of the cell wall by catalyzing dephosphorylation of α-xylidase and β-glucosidase in the cell wall (Kaida et al., 2009). GRMZM2G136971 encodes leucine-rich repeat extensin-like protein 3. Baumberger et al. (2001) found *LRX1*, a new Arabidopsis gene that encodes a chimeric leucine-rich repeat/extensin protein. At the same time, their results suggested that LRX1 was a potential regulator of cell wall development.

Two candidate genes were found to be related to the mechanical strength of stem. GRMZM2G167520 encoded brittle stalk-2-like protein 6, and GRMZM2G167497 encoded brittle stalk-2-like protein 7. Ching et al. (2006) showed that the expression of *Brittle stalk 2* (*BK2*) genes in maize stem, root, and leaf tissue affected the mechanical strength of maize stem. However, the expression was the highest in the vascular systems. The role of the *BK2* genes in secondary wall formation is consistent. All in all, further studies are needed for the functional validation of these candidate genes to discover the possible mechanism of vascular bundle regulation.

## Data Availability Statement

The original contributions presented in the study are included in the article/Supplementary Material, and further inquiries can be directed to the corresponding authors.

## Author Contributions

HW and JG conceived the ideas, designed the methodology, and revised the manuscript. YZ conducted the field trials and collected the data. PH, LZ, WS, HL, and YH took part in experiment and analyzed the data. YZ and PH wrote the manuscript. All authors have read and approved the final manuscript.

### Conflict of Interest

The authors declare that the research was conducted in the absence of any commercial or financial relationships that could be construed as a potential conflict of interest.

## Footnotes

^1^ https://www.ncbi.nlm.nih.gov/

^2^ http://www.geneontology.org/

^3^ http://kobas.cbi.pku.edu.cn/kobas3/?t=1

^4^ www.maizegdb.org
